# Post-operative pain control in arthroscopic rotator cuff repairs: a prospective, double-blinded, randomized controlled trial comparing interscalene catheters and single-shot blocks

**DOI:** 10.1016/j.jseint.2026.101661

**Published:** 2026-02-11

**Authors:** David Gamble, Jay R. Ebert, Sam Shales, Brad Lawther, Travis M. Falconer

**Affiliations:** a Department of Trauma and Orthopaedic Surgery, Sir Charles Gairdner Hospital, Perth, Western Australia, Australia; b Perth Orthopaedic and Sports Medicine Research Institute, Perth, Western Australia, Australia; c School of Human Sciences (Exercise and Sports Science), University of Western Australia, Perth, Western Australia, Australia; d Perth Orthopaedic and Sports Medicine Centre, Perth, Western Australia, Australia

**Keywords:** Arthroscopic rotator cuff repair, Single-shot block, Interscalene block, Continuous interscalene catheter, Opioid use, Pain management

## Abstract

**Background:**

Post-operative pain following arthroscopic rotator cuff repair is challenging. Peripheral nerve blocks are commonly used, but debate remains over single-shot versus continuous interscalene catheters. This trial compared early post-operative pain and opioid use with a single-shot block versus a continuous interscalene catheter.

**Methods:**

In this prospective, double-blinded randomized controlled trial, 45 patients undergoing arthroscopic rotator cuff repair received an interscalene catheter with an initial single-shot block. They were randomized to either a patient-controlled infusion of normal saline (control, n = 22) or low-dose anesthetic (treatment, n = 23). Visual analogue scale for pain severity (VAS-S) and frequency (VAS-F) were assessed pre-operatively, daily for the first post-operative week, and at 2, 6, and 12 weeks. Opioid consumption was recorded for one week post-operatively. Functional outcomes were measured using the Constant and Western Ontario Rotator Cuff scores, and satisfaction with the surgery was assessed at 12 weeks.

**Results:**

A significant interaction effect was found for VAS-S over the first week (*P* = .041), with the treatment group reporting significantly lower pain on day 1 (*P* = .011). Both groups showed significant improvement in clinical scores (*P* < .0001). The treatment group had a higher Constant score at 12 weeks (*P* = .040), though this did not reach the Minimally Clinically Important Difference. No significant difference in opioid consumption (*P* = .653) or satisfaction (treatment: 82%, control: 91%) was observed.

**Conclusion:**

Continuous interscalene catheters improved early post-operative pain without reducing opioid use. Despite these findings, careful consideration of patient needs, cost-effectiveness, and the potential complications associated with catheter use should be acknowledged in the individual patient's pain management strategy.

Rotator cuff pathology is common, with a 200-400% reported increase in the number of arthroscopic rotator cuff repairs (RCRs) being performed and an increasing trend toward the procedure being performed as a short stay or outpatient basis.^39^ Shoulder surgery has long been associated with post-operative pain, especially in the early stages of recovery.^31^^,^^34^ Effective post-operative pain relief following arthroscopic RCR is important for ensuring optimal recovery, rehabilitation, and patient satisfaction.^20^^,^^33^^,^^38^

Peripheral nerve blocks have emerged as a vital tool in the multimodal analgesic strategy in post-operative care.^3^ They are frequently utilized to provide enhanced analgesia while aiming to minimize the reliance on narcotic medications.^37^ The reduction in narcotic use is particularly important in mitigating the risk of various adverse effects such as nausea, vomiting, constipation, and ileus, which can complicate recovery and predispose to early patient dissatisfaction.^10^, ^11^, ^12^

The interscalene block (ISB) is the most employed technique in shoulder and upper extremity surgeries.^2^^,^^3^ The ISB can be primarily administered in 2 ways: as a single-shot block (SSB) or through a continuous indwelling catheter (CIC). The SSB technique involves a one-time injection, which provides immediate pain relief but may have a shorter duration of effect. Conversely, the CIC method allows for continuous administration of local anesthetic, aiming to provide longer-lasting pain management in the immediate post-operative period.

Compared to general anesthesia, regional anesthesia has been shown to be safe, providing excellent pain relief and reducing hospital stays.^25^ Studies have demonstrated increased patient satisfaction and outcomes when comparing continuous ISBs and SSB.^40^ Despite these benefits, many surgeons continue to prefer the SSB method in outpatient settings. In a survey conducted among members of the American Shoulder and Elbow Surgeons, the majority (58.7%) indicated that they would choose a single-shot ISB for themselves.^26^ This preference often stems from concerns regarding higher complication rates associated with catheter use, such as infection, catheter migration, or irritation at the injection site.^24^ Irrespective of the technique, surveyed surgeons remain concerned about rebound pain, which can be a major source of morbidity for patients.^7^

This study sought to investigate early post-operative pain, narcotic requirements, and shoulder function in patients undergoing arthroscopic RCR repair with an interscalene SSB or CIC. It was hypothesized that patients receiving CIC would report lower early post-operative pain, resulting in lower opioid use and higher patient satisfaction.

## Materials and methods

The Consolidated Standards of Reporting Trials reporting guideline for randomized controlled trials (RCTs) was followed.^30^

### Study design

This prospective, double-blinded, RCT investigated early patient outcomes in patients undergoing arthroscopic RCR with a CIC or SSB. Ethics approval was obtained through the relevant hospital Human Research Ethics Committee. Prior to recruitment, a detailed informed consent process was conducted by the senior author of the paper, ensuring patients understood the risk and benefits of the procedure and the regional anesthetic options. All patients received an institutional approved written information sheet in compliance with research trial guidelines prior to final consent.

### Participants

**Figure 1:**
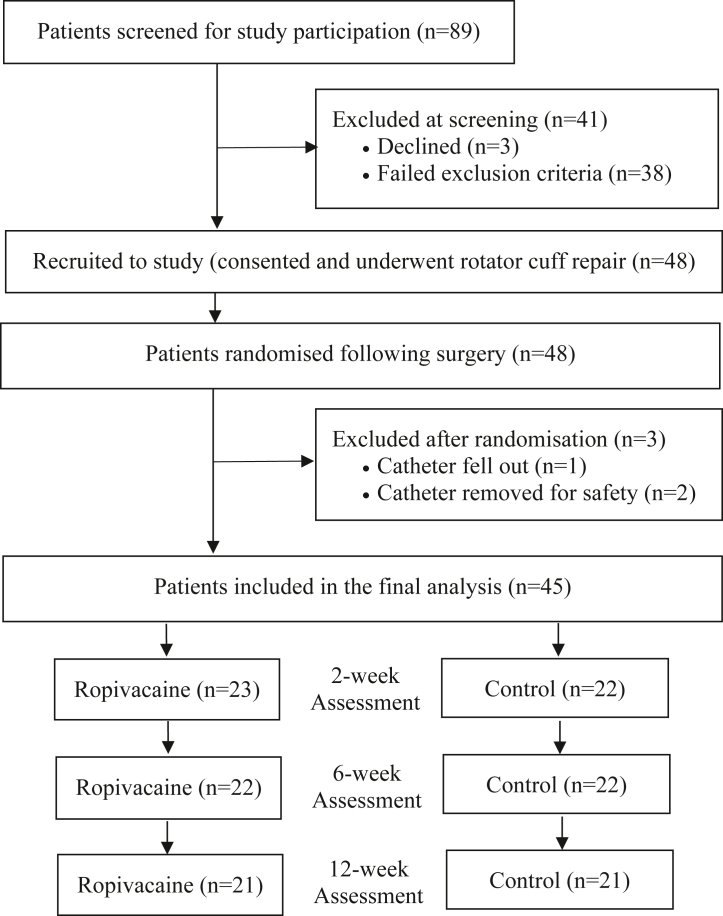
CONSORT flowchart demonstrating patient screening, recruitment, randomization, evaluation, and loss to follow-up over the study period. *CONSORT*, Consolidated Standards of Reporting Trials.

All patients attending the private orthopedic clinic of a single surgeon with symptomatic supraspinatus tears between November 2019 and January 2024 were screened for eligibility (Fig. 1). Patients were considered eligible for enrollment if they met the following criteria: ≥18 years of age, diagnosed with an isolated supraspinatus tear both clinically and radiologically on magnetic resonance imaging, and scheduled for arthroscopic RCR given persistent symptoms and failed nonoperative management including physiotherapy. Exclusion criteria included inability to speak or read English, previous surgery to the ipsilateral shoulder, other concurrent shoulder pathology (massive cuff tears, subscapularis tears, infraspinatus tears, and patients requiring sub pectoralis biceps tenodesis), surgery under the workers' compensation scheme, previous history of fibromyalgia, symptomatic cervical radiculopathy, brachial plexopathy, chronic regional pain syndrome, or a known history of opioid dependence.

### Interventions

The surgery was performed by a single fellowship-trained senior consultant upper limb surgeon, while all regional blocks were carried out by a single consultant anesthetist specializing in regional anesthesia. At the time of surgery, all patients underwent a general anesthetic and received an interscalene catheter inserted under ultrasound guidance. All patients then received a single-shot 15 ml bolus dose of 0.2% ropivacaine.

The surgical technique was standardized for all patients. All patients were positioned in a beach chair for the procedure. After a standard diagnostic shoulder arthroscopy, an arthroscopic double-row RCR with knotless nonabsorbable anchors with suture tape was employed in all cases. Two 3.5 mm PushLock (Arthrex, Naples, FL) pre-loaded with FiberTape (Arthrex) and TigerTape (Arthrex) used as medial row and passed using a Speedbridge repair into two 5.5 mm SwiveLock (Arthrex) lateral row anchors. Acromioplasty was performed in all patients, and a biceps tenotomy or tenodesis was performed if required. Tenodesis was done arthroscopically to the lateral row with 2 FiberWire (Arthrex) suture passed through the long head of the biceps tendon in required cases to avoid using additional anchors.

### Randomization

Patients were randomized in a 1:1 ratio to 1 of 2 groups: 1) patient-controlled infusion catheter filled with normal saline (SSB, control group), or 2) patient-controlled infusion pump filled with low-dose anesthetic (CIC, intervention group). Group randomization occurred following the completion of surgery in the recovery room after the interscalene catheter had been inserted. Patients were reviewed in recovery by the Acute Pain Service, and the Lead Nurse would open a sealed opaque envelope revealing which group the patient was to be randomized to. Participants and researchers were both blinded to the allocation. The SSB (control) group received an infusion of 0.9% normal saline disguised in a “sham” bag labeled identically as the study arm. The CIC (intervention) group received an infusion of 0.2% ropivacaine 2 ml/h and 5 ml breakthrough boluses.

Post-operatively, all patients received the same prescribed regular and breakthrough analgesia. This was 7 days of regular 50 mg slow release Tapentadol twice per day, with 50-100 mg immediate release as needed for breakthrough pain. Patients were discharged on day 1, and catheters were removed by the patients following a phone call by the pain nurse on day 3. Post-operative rehabilitation was standardized for both groups with a sling and gentle pendular exercises for 6 weeks, followed by a 6-week period of weaning from the sling and commencement of assisted active range of motion exercises and strengthening initiated after 12 weeks.

## Outcomes

### Primary outcome

The primary outcome of this study was severity of pain via a visual analog scale for pain severity (VAS-S), scored from 0 (no pain) to 10 (extreme pain). Patients were asked to score the worst pain they had experienced that day. This was assessed every day for the first post-operative week and at 2, 6, and 12 weeks postsurgery. Scores were recorded via a logbook prospectively entered by the patients.

### Secondary outcomes

Secondary outcomes included a visual analogue scale for pain frequency (VAS-F) ranging from 0 (never) to 10 (constant pain). Patients were asked how often they had pain that week and scored from 0 (never) to 10 (constant) and this was recorded prospectively in a logbook by the patients. Other secondary outcomes were the Western Ontario Rotator Cuff (WORC) score and Constant score. Minimally Clinically Important Difference (MCID) for the Constant score was defined at 10.4^23^. The 10.4 MCID for the Constant score is consistent with values previously established through anchor-based analyses in comparable populations undergoing RCR.^23^ These secondary subjective outcomes were collected pre-operatively and at 12 weeks postsurgery. The WORC (0-100) was employed to assess pain, symptoms, sport/recreation, and work function, as well as lifestyle and emotional variables.^21^ The Constant score (0-100) was calculated from individual subdomains of patient-reported pain (0-15 points), the effect of the shoulder condition on occupational, leisure, and other daily activities (0-20 points), active range of motion (0-40 points), and maximal pain-free isometric abduction strength in 90° of shoulder abduction in the scapular plane (0-25 points).^5^^,^^6^ Patient satisfaction with the surgical outcome was assessed at 12 weeks postsurgery using a 4-point Likert response scale with descriptors very satisfied, somewhat satisfied, somewhat dissatisfied, and very dissatisfied. Finally, opioid usage was collected prospectively in a logbook each day for the first post-operative week and converted to milligram of morphine equivalent for standardization.

### Sample size

For this prospective RCT, a *priori* sample size power calculation was determined employing G-Power (Dusseldorf, Germany). The primary outcome variable was the post-operative VAS severity score on day 1 postsurgery. Anticipating a large effect of size (d = 0.80) in shoulder pain between the 2 analgesic pathways, an estimated sample of 52 (26 per group) was initially required to reveal differences at alpha 0.05 with 80% power.

### Statistical methods

The mean (standard deviation [SD]) of all pre- and post-operative clinical scores were calculated and presented. Normality of distribution of continuous data was assessed and confirmed via the Shapiro-Wilk test. Repeated measures analysis of variance was employed to investigate differences between groups over time, with post hoc tests in the presence of significant group effects. For the 2 groups, the number (and percentage) of patients reporting “very satisfied,” “somewhat satisfied,” “somewhat dissatisfied,” and “very dissatisfied” at 12 weeks was presented. Where appropriate, statistical analysis was performed using SPSS software (SPSS, Version 29.0, SPSS Inc., USA), with significance determined at *P* < .05.

## Results

This study recruited 48 patients who subsequently underwent arthroscopic RCR between November 2019 and January 2024. Eighty-nine patients met the inclusion criteria and were screened for the trial. Forty-one patients were excluded following screening, giving a participation rate of 54%. Of the 48 patients recruited, 45 patients (intervention = 23, control = 22) were included immediately following surgery and in the final analysis (Fig. 1).

No group differences were observed in key demographics between the control and treatment arms, including age (*P* = .761), gender distribution (*P* = .709), and whether the operated limb was the dominant limb (*P* = .741) (Table I). Biceps procedures were similarly distributed between groups (*P* = .619), with biceps tenodesis performed in 5 of 22 patients (22%) in the intervention group and 7 of 23 patients (30%) in the control group. Due to this comparable distribution, biceps management was not included as a covariate in the statistical analysis.

**Table I:** Participant demographics in patients retained following surgery and randomization, for both the control and intervention groups.

Variable	Intervention (ropivacaine) n = 23	Control (saline) n = 22	*P* value
Mean age (yr)	56.9 ( ± 12)	58.2 ( ± 15.1)	.761
Sex – male n (%)	17 (71%)	18 (75%)	.709
Dominant limb – operated n (%)	15 (63%)	16 (67%)	.741

**Figure 2:**
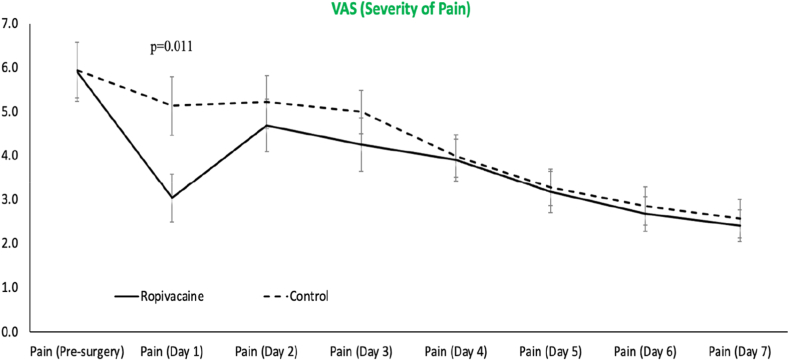
Progression of patient-reported VAS-S for both the control and intervention groups over the first 7 post-operative days. *VAS-S*, visual analogue scale for pain severity.

A significant interaction effect (*P* = .041) was observed over the first seven days for the VAS-S, with post hoc assessment indicating a lower severity of pain on day 1 postsurgery in the treatment group (*P* = .011) (Fig. 2). However, for the VAS-S and VAS-F collected at 2, 6, and 12 weeks postsurgery, no group differences were observed (*P* > .05). All clinical scores (in both groups) significantly improved (*P* = .0001) from presurgery to postsurgery (Table II). A significant group effect (*P* = .015) was observed for the Constant score, with the 9.2-point difference between groups statistically significant (*P* = .040). However, both groups showed clinically meaningful improvement in Constant scores, with the CIC group improving by 20.2 points and the SSB group by 15.3 points. These both exceeded the MCID of 10.4. No group differences were also observed for total WORC and WORC subscales pre-operatively and at 12 weeks (Table II). No statistically significant difference in opioid consumption (*P* = .653) between the 2 groups was observed (Table II). At 12 weeks, 82% of the treatment group were satisfied with their treatment outcome, compared to 91% of the control group (Table III). While one recruited patient was excluded from the study as the catheter had fallen out, there were no observed surgical or post-operative complications encountered, as well as no other complications encountered with the analgesic treatment pathways.

**Table II:** Summary of PROMs and opioid use for both the control and intervention groups.

Variable or PROM	Intervention group (n = 23)	Control group (n = 22)
Mean VAS-S (SD)		
Pre-surgery	5.1 (2.5)	5.5 (2.4)
2 weeks	3.7 (2.1)	4.0 (2.6)
6 weeks	2.7 (2.1)	3.2 (2.4)
12 weeks	2.2 (2.1)	2.6 (2.1)
Mean VAS-F (SD)		
Pre-surgery	5.8 (3.1)	6.2 (2.6)
2 weeks	3.5 (2.4)	3.1 (2.3)
6 weeks	2.7 (2.4)	2.5 (2.2)
12 weeks	2.4 (2.2)	2.5 (2.2)
Opioid use		
Morphine milligram equivalents (MME)	269 (167)	295 (222)
Mean Constant score (SD)		
Pre-surgery	55.3 (20.5)	51.0 (18.4)
12 weeks	75.5 (11.7)	66.3 (11.6)
Mean pre-surgery WORC scores (SD)		
Total	44.6 (21.7)	43.2 (17.0)
Physical symptoms	51.5 (21.0)	52.0 (15.9)
Sports recreation	34.9 (20.7)	31.5 (18.0)
Work	33.5 (25.6)	34.1 (17.6)
Lifestyle	51.9 (25.7)	44.4 (19.4)
Emotion	49.1 (29.8)	46.5 (28.1)
Mean post-surgery WORC scores (SD)		
Total	72.2 (16.7)	70.5 (16.6)
Physical symptoms	83.8 (13.8)	77.6 (15.3)
Sports recreation	48.4 (20.4)	52.6 (29.2)
Work	65.1 (28.2)	61.8 (22.3)
Lifestyle	78.3 (19.1)	79.1 (18.1)
Emotion	84.5 (21.7)	80.2 (21.1)

*WORC*, Western Ontario Rotator Cuff; *PROMs*, patient-reported outcome measures.

**Table III:** Post-operative patient satisfaction for both the control and intervention groups.

Satisfaction score	Intervention group (ropivacaine) n = 23	Control group (saline) n = 22	*P* value
Very satisfied	14	15	-
Somewhat satisfied	4	5	-
Somewhat dissatisfied	1	1	-
Very dissatisfied	0	0	-
Did not complete	3	1	-
Overall satisfied (%)	18 (82%)	20 (91%)	.380

## Discussion

The most important findings of the current study were that patients undergoing arthroscopic RCR followed by a CIC, versus SSB, reported less early post-operative severity of shoulder pain based on the VAS-S score. However, no differences in other scores were observed, including the requirement for opioids.

While the CIC group reported a significantly lower severity of pain on the first post-operative day, the results of the current study otherwise demonstrated similar pain profiles between the CIC and SSB groups. As previously mentioned and other studies have suggested,^1^ this difference may be attributed to rebound pain, particularly in the SSB group, where the block duration typically lasts 8–12 hours.^40^ Interestingly, the CIC group did not show an equivalent rebound pain pattern by day 4, once the catheter had been removed. A multimodal approach, including regional blocks, has been shown to provide significant pain relief and improve patient satisfaction in arthroscopic shoulder surgery.^34^ This approach becomes especially relevant given the growing trend toward outpatient and same-day discharge for shoulder surgeries.^25^^,^^29^^,^^36^

Despite a trend toward lower opioid consumption in the CIC group, no statistically significant difference was observed between the 2 groups. Interestingly, this finding contradicts existing literature^16^^,^^22^^,^^24^^,^^29^ which has shown that narcotic consumption is lower in patients receiving CIC, particularly in the immediate post-operative period. Malik et al^25^ reported reduced narcotic use in a CIC group following arthroscopic RCR during the first 3 post-operative days, while Salviz et al^29^ also reported decreased narcotic use at Day 1 and 2 in a CIC group. The reported opioid crisis^9^^,^^18^^,^^28^^,^^37^ highlights the urgent need to balance effective pain management with the risks of opioid dependence and misuse. Despite this, we found no significant reduction in opioid consumption between the 2 groups over the first week. A limitation in our study design was that the combination of conducting an ethical pain study whilst having effective blinding with a sham saline bag for the control group required use of a standardized post-operative analgesia regimen prescribed for all patients. The prescription of regular sustained release tapentadol potentially inflated overall opioid consumption making it harder to elicit statistical significance. Regardless, both treatment arms resulted in high patient satisfaction, reflecting the overall efficacy of both analgesic strategies.

An unexpected finding in this study was the statistically significant improvement in Constant scores at 12 weeks in the CIC group compared to the SSB group. This indicates that both analgesic pathways were associated with meaningful functional recovery. Although the between-group difference of 9.2 points reached statistical significance, it did not surpass the MCID threshold, suggesting limited clinical relevance.^23^ Both groups achieved meaningful functional recovery, and opioid consumption did not differ significantly. Thus, while the CIC technique may offer a modest incremental benefit, its routine use should be carefully considered in light of the additional procedural complexity, resource utilization, and potential risks associated with an indwelling catheter. A possible explanation for the improvement is that by optimizing pain control early in the post-operative phase and preventing day 1 rebound pain, patients may have been better able to engage in rehabilitation. In our institution, the first physiotherapy session occurs on day 1 post-op, coinciding with the surgical follow-up visit, where the rehabilitation regimen is outlined. While post-operative rehabilitation in the current study was standardized across both groups, rehabilitation adherence was not. Nonetheless, post-operative pain is known to cause impaired cognition, hindering memory and rehabilitation compliance.^4^^,^^19^ Optimizing pain control early on at this crucial phase may have facilitated greater adherence to rehabilitation protocols. This concept has been supported by studies investigating prehabilitation in both the shoulder and lower limb joint arthroplasty fields, where the implementation of pre-operative "joint schools" has proven effective in improving post-operative outcomes and patient satisfaction.^13^^,^^14^^,^^35^

Although the observed difference in satisfaction between groups did not reach statistical significance, the trend toward higher satisfaction in the SSB group—despite comparable analgesic outcomes—warrants consideration. Patient satisfaction is multifactorial and influenced not only by analgesic efficacy but also by expectations, convenience, and the perceived invasiveness and maintenance burden of the intervention. For example, catheter-based techniques may carry a perceived or real burden related to catheter care, mobility limitations, or device anxiety, whereas a single-shot approach may be valued for its simplicity and lower maintenance demands. Importantly, both groups' satisfaction levels are consistent with recent literature^15^ reporting high overall satisfaction with peripheral regional analgesia.

An SSB may be a more economical option for providing sufficient analgesia in RCR, as it reduces the need for continuous infusion catheter pumps, which can incur additional costs and complications. A large cost analysis by Jones et al^17^ found that the material cost of CIC is approximately $450 higher than SSB. Furthermore, CIC requires additional personnel, such as nurses or clinical staff, to monitor patients post-operatively. A recent study on shoulder arthroplasty also revealed that CIC was associated with longer hospital stays and potential discharge delays.^32^ In addition to these costs, the time and resources spent on training staff to manage catheter pumps, as well as addressing any mechanical issues with the pumps, must also be factored into the overall cost analysis.^27^ Importantly, none of the patients in this cohort experienced major complications related to the SSB or CIC. Although this RCT was not designed to evaluate complication rates, existing literature suggests that minor complications such as erythema and bruising at the catheter insertion site may occur in up to 48% of cases.^24^ The clinical significance of these minor events remains unclear.

Several study limitations should be acknowledged. Firstly, the potential statistical frailty associated with the relatively small sample size is acknowledged. The current trial was designed as a pragmatic RCT within the constraints of a single-surgeon, single-anesthetist setting. The trial was powered using the primary aim of detecting an anticipated large effect size in early post-operative VAS severity scores on day 1 post-surgery, though it is unlikely the sample was therefore adequately powered to detect group-based differences in other outcomes and at later post-operative follow-up time points. This approach was selected not only based on ethical and logistical feasibility but also in line with prior literature suggesting that meaningful clinical improvements in acute pain scores typically reflect large effect sizes. Secondly, to maintain ethical standards, ensure effective blinding, and provide satisfactory post-operative pain control, both groups were managed as if they had active catheters, thereby minimizing the risk of rebound pain once the initial block wore off. All patients also received standardized post-operative opioid analgesia, including extended-release opioids, which may have confounded opioid consumption data and potentially attenuated between-group differences. Both groups’ total morphine milligram equivalents (MME) is in keeping with recent published literature for MME^8^ following shoulder surgery; however, a high mean MME is a potential limitation for assessing the analgesic effects of regional anesthesia. Thirdly, patient-reported pain scores are susceptible to recall and response bias. Finally, the generalizability of the study findings may be limited due to this being a single-center study involving a single surgeon and anesthetist and only incorporating isolated supraspinatus RCR. However, this also ensures the standardization of treatment across all patients. Further to this and in support of the study, true blinding was maintained through a well-constructed trial design with prospective randomization and the use of “sham” saline bags. Furthermore, the current study comprehensively tracked the recovery of pain and function over a full 12-week follow-up period. The study had a high participation rate of 54% and a low dropout rate of 6% following randomization which reduced the risk of selection bias and improved the generalizability of the study.

## Conclusion

This study supports the safe and effective use of CIC for post-operative pain management following arthroscopic RCR, with patients undergoing a CIC (versus SSB) analgesic pathway reporting less early post-operative severity of shoulder pain without any observed complications. However, no differences in other scores were observed, including the requirement for opioids. Overall, the current study highlights the marginal benefit of CIC compared to SSB. Given the similar analgesic outcomes and the added costs and potential complications associated with CIC (albeit not observed in the current study), these findings underscore the importance of evidence that helps refine clinical indications, promote cost-effective care, and limit unnecessary invasive procedures.

## Disclaimers:

Funding: An independent research grant was awarded by the Hollywood Private Hospital Research Foundation (RF113), while institutional support was provided by the Perth Orthopaedic and Sports Medicine Research Institute.

Conflicts of interest: The authors, their immediate families, and any research foundations with which they are affiliated have not received any financial payments or other benefits from any commercial entity related to the subject of this article.
